# Impact of Community-Wide Tuberculosis Active Case Finding and Human Immunodeficiency Virus Testing on Tuberculosis Trends in Malawi

**DOI:** 10.1093/cid/ciad238

**Published:** 2023-04-26

**Authors:** Rachael M Burke, Marriott Nliwasa, Peter J Dodd, Helena R A Feasey, McEwen Khundi, Augustine Choko, Rebecca Nzawa-Soko, James Mpunga, Emily L Webb, Katherine Fielding, Peter MacPherson, Elizabeth L Corbett

**Affiliations:** Malawi Liverpool Wellcome Clinical Research Programme, Blantyre, Malawi; Clinical Research Department, Faculty of Infectious and Tropical Diseases, London School of Hygiene and Tropical Medicine, London, United Kingdom; Malawi Liverpool Wellcome Clinical Research Programme, Blantyre, Malawi; Helse Nord Tuberculosis Initiative, Kamuzu University of Health Sciences, Blantyre, Malawi; School of Health and Related Research, University of Sheffield, Sheffield, United Kingdom; Malawi Liverpool Wellcome Clinical Research Programme, Blantyre, Malawi; Clinical Research Department, Faculty of Infectious and Tropical Diseases, London School of Hygiene and Tropical Medicine, London, United Kingdom; Malawi Liverpool Wellcome Clinical Research Programme, Blantyre, Malawi; (MRC) International Statistics and Epidemiology Group, Faculty of Epidemiology and Population Health, London School of Hygiene and Tropical Medicine, London, United Kingdom; Malawi Liverpool Wellcome Clinical Research Programme, Blantyre, Malawi; Malawi Liverpool Wellcome Clinical Research Programme, Blantyre, Malawi; National Tuberculosis Programme, Government of Malawi, Lilongwe, Malawi; (MRC) International Statistics and Epidemiology Group, Faculty of Epidemiology and Population Health, London School of Hygiene and Tropical Medicine, London, United Kingdom; (MRC) International Statistics and Epidemiology Group, Faculty of Epidemiology and Population Health, London School of Hygiene and Tropical Medicine, London, United Kingdom; Malawi Liverpool Wellcome Clinical Research Programme, Blantyre, Malawi; Clinical Research Department, Faculty of Infectious and Tropical Diseases, London School of Hygiene and Tropical Medicine, London, United Kingdom; School of Health and Wellbeing, University of Glasgow, Glasgow, United Kingdom; Clinical Research Department, Faculty of Infectious and Tropical Diseases, London School of Hygiene and Tropical Medicine, London, United Kingdom

**Keywords:** epidemiology, tuberculosis, HIV, active case finding

## Abstract

**Background:**

Tuberculosis case-finding interventions are critical to meeting World Health Organization End TB strategy goals. We investigated the impact of community-wide tuberculosis active case finding (ACF) alongside scale-up of human immunodeficiency virus (HIV) testing and care on trends in adult tuberculosis case notification rates (CNRs) in Blantyre, Malawi.

**Methods:**

Five rounds of ACF for tuberculosis (1–2 weeks of leafleting, door-to-door enquiry for cough and sputum microscopy) were delivered to neighborhoods (“ACF areas”) in North-West Blantyre between April 2011 and August 2014. Many of these neighborhoods also had concurrent HIV testing interventions. The remaining neighborhoods in Blantyre City (“non-ACF areas”) provided a non-randomized comparator. We analyzed TB CNRs from January 2009 until December 2018. We used interrupted time series analysis to compare tuberculosis CNRs before ACF and after ACF, and between ACF and non-ACF areas.

**Results:**

Tuberculosis CNRs increased in Blantyre concurrently with start of ACF for tuberculosis in both ACF and non-ACF areas, with a larger magnitude in ACF areas. Compared to a counterfactual where pre-ACF CNR trends continued during ACF period, we estimated there were an additional 101 (95% confidence interval [CI] 42 to 160) microbiologically confirmed (Bac+) tuberculosis diagnoses per 100 000 person-years in the ACF areas in 3 and a half years of ACF. Compared to a counterfactual where trends in ACF area were the same as trends in non-ACF areas, we estimated an additional 63 (95% CI 38 to 90) Bac + diagnoses per 100 000 person-years in the same period.

**Conclusions:**

Tuberculosis ACF was associated with a rapid increase in people diagnosed with tuberculosis in Blantyre.

## Teaser

Tuberculosis active case finding (ACF) in Blantyre, Malawi, was associated with increased tuberculosis case notifications 2011–2014, in interrupted time series analysis. Most of the increase in tuberculosis notifications came from routine health system, not direct from ACF sputum samples.

## Graphical abstract

**Figure:**
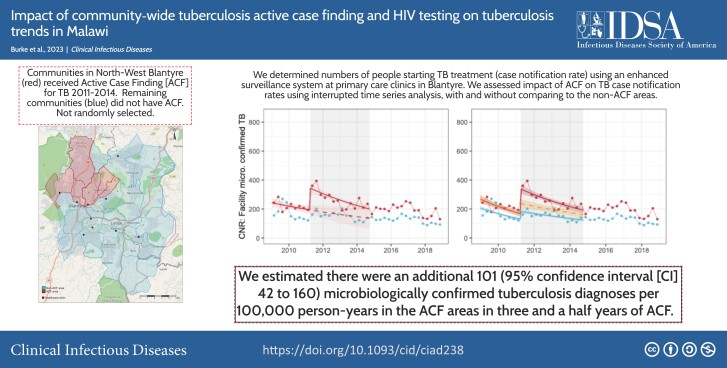
This graphical abstract is also available at Tidbit: https://tidbitapp.io/tidbits/impact-of-community-wide-tuberculosis-active-case-finding-and-hiv-testing-on-tuberculosis-trends-in-malawi-b1fce306-ce6e-401d-ae7e-88d63dd4b4ae

**(See the Editorial Commentary by Chaisson on pages 101–2.)**

Worldwide, an estimated 10 million people developed tuberculosis disease in 2020. However, 4 million of these people were either not diagnosed or were diagnosed but not notified through national reporting systems [1]. People with undiagnosed or untreated tuberculosis disease can transmit tuberculosis to others in their households and communities and are at risk of severe illness and death [2]. National tuberculosis prevalence surveys in several African countries demonstrate high burdens of undiagnosed tuberculosis disease, indicative of long diagnostic delays [3–5].

Adding community-based active case finding (ACF) interventions to facility-based services can increase the number of number of people diagnosed with tuberculosis, with some—but not all—studies reporting reductions in tuberculosis prevalence [6–10]. Community-based ACF in populations with a high prevalence of undiagnosed disease is one of the approaches recommended by the World Health Organization (WHO). Ideally, ACF should be combined with scale-up of human immunodeficiency virus (HIV) testing and antiretroviral treatment (ART) services, and strengthening of facility-based tuberculosis diagnostics and infection control, with combined interventions most likely to maximize and maintain individual and public-health benefits from early tuberculosis diagnosis [11].

The aim of this study was to investigate the immediate and longer-term impacts of ACF on trends of tuberculosis case notifications rates in urban Blantyre, Malawi during a time of successful national ART scale-up. We estimated the number of increased tuberculosis diagnoses due to ACF, compared to counterfactual scenarios constructed from trends of tuberculosis diagnoses in non-ACF areas and in pre-ACF time periods.

## METHODS

### Setting and Study Area Demarcation

**Figure 1.**
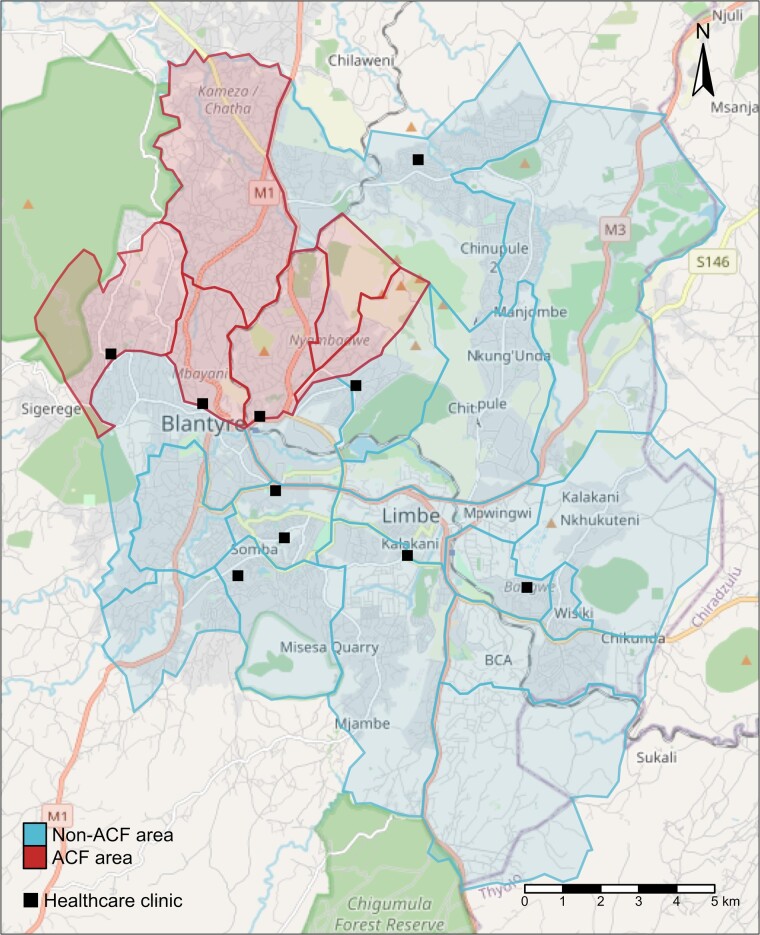
Map of urban Blantyre showing administrative district boundaries and ACF and non-ACF areas. Abbreviation: ACF, active case finding.

Community-based active tuberculosis case finding was conducted in high density residential suburbs in North-West Blantyre (“ACF areas”) between April 2011 and October 2014. The ACF areas were selected on basis of high levels of poverty, poor access to health services, and also physical proximity to the Kamuzu University of Health Sciences tuberculosis laboratory. The remainder of urban Blantyre were designated as “non-ACF” neighborhoods (Figure 1). The “non-ACF” areas included more affluent areas and the central business district, as well as high density residential suburbs with similar levels of poverty as the ACF areas (but further from the tuberculosis lab). The estimated adult (age ≥15 years) population of “ACF” and “non-ACF” areas in 2011 was 130 173 and 290,961, respectively. People living in rural areas outside the city (“Blantyre Rural” district) or in other districts were excluded from this analysis, even if they received a tuberculosis diagnosis from a health facility in Blantyre City. Estimated Blantyre adult HIV prevalence was 16.7% in March 2016 [12].

### Active Tuberculosis Case Finding Intervention

Five rounds of community-based ACF were conducted in the ACF area suburbs between April 2011 and August 2014. Twenty-three zones were visited for 1–2 weeks at a time by community workers approximately every 8 months. Activities included megaphone announcements, establishing a central point within the zone, and door to door enquiry for cough. For any household where an adult (present or not) had a cough, 2 sputum pots were left and collected the following morning [12]. Limitations of sputum microscopy were discussed with community members at the time of sputum collection and results, with signposting to primary care clinics if persistent symptoms. We did not specifically do any ACF activities in healthcare centers, other than training TB officers to record cases for enhanced TB surveillance system.

### Tuberculosis and HIV Services

All health facilities had smear microscopy available for tuberculosis diagnosis throughout this period. Xpert MTB/RIF was introduced from January 2014. Chest X-ray was typically not available in health facilities, although from March 2018 onward the National Tuberculosis Programme provided some mobile X-ray services. Facility-based HIV testing was available, and ART was available to people living with HIV with low CD4 cell counts, pregnancy, or WHO stage 3 or 4 defining conditions (see Appendix) and to all people living with HIV from July 2016 onward. Within the ACF area during 2011–2014, approximately one third of adults also had access to community-based HIV self-testing and linkage to care interventions (including home-based ART), provided by the research team as part of a trial [13].

### City-wide Electronic Registration of People Starting Tuberculosis Treatment

We established an enhanced tuberculosis surveillance system in 2011. From March 2011 to 2015, tuberculosis officers completed a notification form for each person starting tuberculosis treatment. From 2011 to 2015 this was a paper form later entered into a computer database, with home location (ACF vs non-ACF) ascertained using a “map book,” which delineated areas of the city and was described previously [14]. From 2015 onward tuberculosis officers entered data directly onto a tablet and location was determined with an electronic geolocator system [15–17]. Data from January 2009 to March 2011 were extracted retrospectively from tuberculosis facility treatment registers and location ascertained with reference to map book areas.

### Tuberculosis Diagnosis Laboratory Procedures

We provided sputum smear microscopy for people accessing tuberculosis diagnosis directly through ACF teams at the University TB laboratory. At the healthcare facilities, from April 2011 (ie, the start of ACF) onward we collected a single sputum from all people starting tuberculosis treatment for smear microscopy and culture. Mycobacterial culture was only performed for people who were at the point of starting tuberculosis treatment; it was not used to make the initial diagnosis of tuberculosis.

### Case Definitions

People with tuberculosis were categorized as “pre-treatment Bac+” if sputum smear microscopy or Xpert tests were positive from healthcare facilities or from diagnostic samples provided to ACF team, and “clinically diagnosed” if there was no microbiological confirmation at the time of starting tuberculosis treatment. During pre-ACF and ACF period almost all pre-treatment Bac + were from sputum smear results, as Xpert was only introduced toward end of 2014.

### Statistical Analysis

The main outcome was trend in quarterly pre-treatment Bac + tuberculosis case notifications per 100 000 person-years. We constructed 2 models to compare the effect of ACF to counterfactual scenarios. In model A, the counterfactual scenario was that pre-ACF trends continued throughout the ACF period. Model A considers the ACF areas only (ie, an interrupted time series analysis, without reference to non-ACF areas) [18]. In model B, the counterfactual scenario was that there was an increase in tuberculosis case notifications in the ACF area of the same magnitude as that seen in the non-ACF areas [19].

The mean number of quarterly tuberculosis diagnoses $\left(R_{c,t}\right)$ in population c∈{ACF,nonACF}

during quarter *t* for each model followed:

**Model A** (fitted to ACF population only)


$$\begin{array}{rl} \log\;(R_{ACF,t}) & =\alpha +\beta \times t+(\alpha_{ACF}+\beta_{ACF}\times t)I_{ACF}(t)\\ & \;+\log(\;population_{ACF,t}) \end{array}$$

**Model B** (fitted to ACF and non-ACF populations)


$$\begin{array}{rl} \log\;(R_{c,t}) & =\alpha^{c}+\beta^{c}\times t+(\alpha_{ACF}{}^{c}+\beta_{ACF}{}^{c}\times t)I_{ACF}(t)\\ & \;+\log(\;population_{c,t}) \end{array}$$

where $I_{ACF}(t)$ is an indicator function that is 1 for during the intervention period and 0 otherwise. In model B, the counterfactual for the ACF population that defines the intervention effect uses the coefficients $\alpha_{ACF}^{nonACF}$ and $\beta_{ACF}{}^{nonACF}$ instead of $\alpha_{ACF}^{ACF}$ and $\beta_{ACF}{}^{ACF}$.

We used these models to predict the mean quarterly tuberculosis CNR for the 14 quarter-years when ACF was ongoing (ie, between April 2011 and September 2014) in the ACF areas with ACF and in the 2 counterfactual without-ACF scenarios. We calculated the difference between the observed CNRs and the expected counterfactual CNRs. We used delta method to estimate 95% confidence intervals (CIs) for the expected differences (Appendix). We used the same methods to estimate total tuberculosis diagnoses, which included both Bac + and clinically-diagnosed tuberculosis. Analysis was done in R version 4.0.3.

### Ethical Considerations

This study was approved by the Ethics Committees of the Malawi College of Medicine (Blantyre, Malawi) and the London School of Hygiene and Tropical Medicine (London, UK).

## RESULTS

### ACF Participation and Direct Yield of ACF Smear-positive Tuberculosis Participants

A total of 7066 adults with tuberculosis symptoms attended the temporary ACF bases over the five rounds of ACF, with a sputum smear-positive diagnostic yield of 2.2% (147/7066)—Table 1.

**Table 1.** Participation in Active Case Finding Intervention and Yield in Smear-positive Tuberculosis Disease (Direct Effect of ACF)

Rounds in ACF Neighborhoods		Number of Adult Residents Targeted^a^	Number of Participants Submitting Sputum	Participants With New Smear Positive TB
	Dates	n	n	n (%)
Round 1	Apr to Oct 2011	131 048	1200	26 (2.2%)
Round 2	March to Aug 2012	134 558	1917	30 (1.6%)
Round 3	Sept to 15 Jul 2013	137 189	1678	34 (2.0%)
Round 4	Jul 2013 to Jan 2014	139 820	1325	26 (2.0%)
Round 5	Jan to Aug 2014	141 576	946	31 (3.3%)
Total	…	…	7066	147 (2.2%)

Abbreviations: ACF, active case finding; TB, tuberculosis.

Estimated population of ACF area of adults ages 15 years and older.

### Tuberculosis Case Notifications in Blantyre

**Figure 2.**
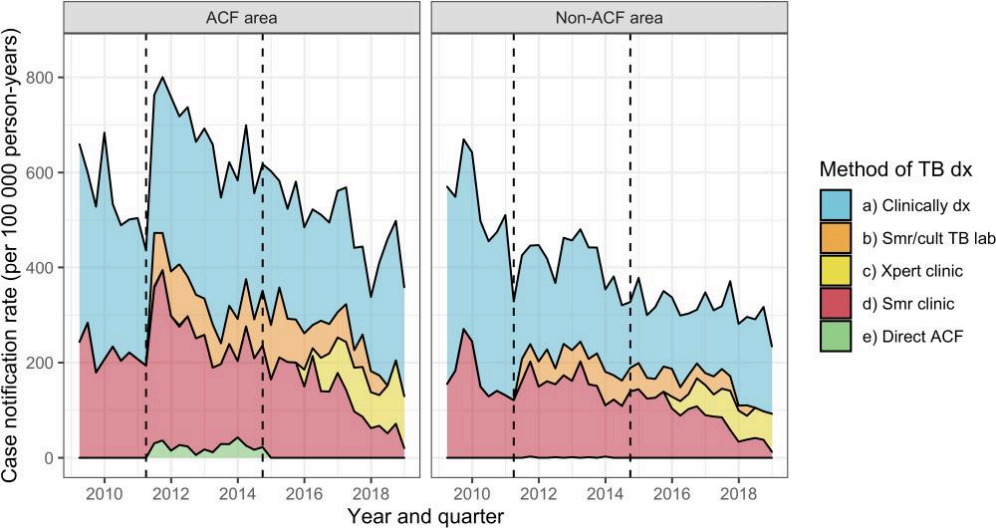
Tuberculosis case notification rates in ACF and Non-ACF areas. (*a*) “Clinically dx” = TB diagnosed without microbiological confirmation (at any time), (*b*) “Smr/cult TB lab” = TB not confirmed microbiologically at the time TB treatment initiation but a sputum sample from taken at the time of TB treatment was subsequently culture or smear positive, (*c*) “Xpert clinic” = Xpert MTB/Rif positive from healthcare facility prior to starting treatment, (*d*) “Smr clinic” = sputum smear microscopy at healthcare facility positive prior to starting treatment, (*e*) “Direct ACF” = Sputum smear positive from a sputum sample collected by the ACF team and processed at the TB research lab. “Pre-treatment Bac+” includes all people in groups (*c*), (*d*), and (*e*). Abbreviations: ACF, active case finding; MTB, *Mycobacteriumtuberculosis*; TB, tuberculosis.

A total of 20 119 adults who lived in Blantyre were diagnosed with tuberculosis (directly through ACF or through services at Blantyre City tuberculosis clinics) and started treatment between 2009 and 2018. Overall (across ACF and non ACF areas), 7598 (37.8%) were pre-treatment Bac + and 12 521 were clinically diagnosed (62.2%)—Figure 2 and Supplementary Table 1.

Individual-level demographic information on people initiating tuberculosis treatment was collected from April 2011 onward. Of the 7613 adults who started tuberculosis treatment from April 2011 to September 2014, 3017 (39.6%) were women, and 4596 (60.4%) were men. The median age was 32 years (interquartile range [IQR] 26–40) among women and 35 years (IQR 29–42) among men. Overall, 4999 people (65.7%) were living with HIV, and 832 (10.9%) had an unrecorded HIV status. Of the 147 people who had tuberculosis diagnosed directly through ACF, 121 registered for tuberculosis treatment and reported living in Blantyre City (all in ACF areas). Nineteen people registered for TB treatment, but reported living outside Blantyre City. Two people died before starting treatment, 2 people moved away from Blantyre before getting results, and 3 people couldn't be traced. Overall, during from April 2011 and September 2014, 4720 people started tuberculosis treatment without microbiological confirmation at the time of starting treatment and 1008 (21.4%) of these had culture confirmed tuberculosis from the sample taken at the start of treatment. The proportion of people whose initial clinical diagnosis of tuberculosis that was culture confirmed was similar in ACF areas (432 / 1,958, 22.1%) and non-ACF areas (576 / 2,762, 20.9%, chi^2^*P* value .33). Supplementary Table 1 contains more details and information on 7625 people who started TB treatment in the “after ACF” period from October 2014 to 2018.

### Trends in Bac + Tuberculosis Case Notification Rates in the ACF Area

**Figure 3.**
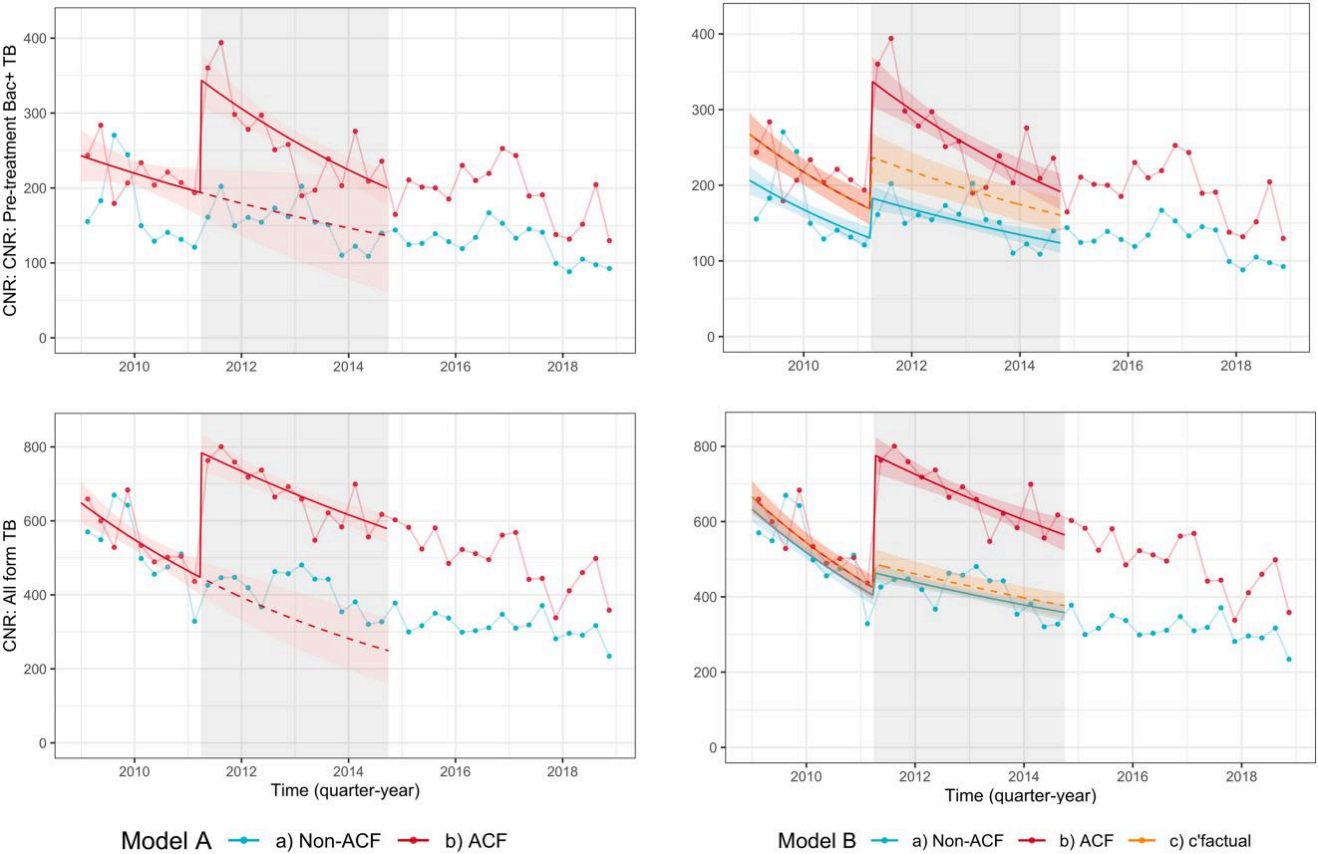
Trends in tuberculosis case notification rates. Quarterly tuberculosis diagnoses per 100 000 person-years (points), with model-predicted tuberculosis diagnoses (solid lines for observed [ACF] scenarios and dotted lines for counterfactual scenarios). Red is ACF areas, blue is non-ACF areas, orange represents a counterfactual scenario where tuberculosis diagnoses increase in ACF area by the same magnitude as they did in non-ACF areas. Top panel is model A (ACF area only) and bottom panel is model B (compared to non-ACF areas). See methods and appendix for model description. Abbreviation: ACF, active case finding.

Pre-treatment Bac + tuberculosis diagnoses by ACF area are shown in Figure 3*A*, together with the model-fitted trends. From 2009 to 2014 (ie, before and during ACF) pre-treatment Bac + tuberculosis is equivalent to smear positive, as Xpert only became available in late 2014. In the 9 quarter-years prior to ACF (ie, January 2009 to March 2011), the mean quarterly pre-treatment Bac + tuberculosis CNRs were 219 and 169 tuberculosis diagnoses per 100 000 person-years in ACF and non-ACF areas, respectively. In the 14 quarter-years where ACF was ongoing (April 2011 to September 2014), mean quarterly pre-treatment Bac + tuberculosis CNRs were 263 and 154 per 100 000 person-years in ACF and non-ACF areas, respectively.

Under model A assumptions (interrupted time series, with no control area), we estimate that, had ACF-area pre-ACF trends in tuberculosis diagnoses continued, there would have been 162 tuberculosis diagnoses per 100 000 person-years in the 14 quarters when ACF interventions were implemented between April 2011 and September 2014. We therefore estimate an absolute increase in mean CNR during the ACF period of 101 tuberculosis diagnoses per 100 000 person-years (95% CI 42 to 160) and a relative increase of 62.2% (95% CI 26.1% to 98.3%)—Table 2.

**Table 2.** Estimated Tuberculosis Diagnoses in ACF Area April 2011 to September 2014 (During ACF) Under Observed and Counterfactual Conditions

…	Mean Quarterly CNR per 100 000 Person-Years; Actual Scenario (ACF)	Mean Quarterly CNR per 100 000 Person-Years; Counterfactual Scenario	Total tuberculosis Diagnosis; Actual Scenario (ACF)	Total tuberculosis Diagnoses; Counterfactual Scenario	Absolute Difference in Mean Quarterly CNR per 100 000 PersonYears	Absolute Difference in Number of Tuberculosis Diagnoses	Relative Difference in CNR per 100 000 Person-Years*
Bac + tuberculosis							
Model A	263	162	1260	775	101 (42 to 160)	480 (198 to 762)	62.2% (26.1%– 98.3%)
Model B	263	199	1260	951	64 (38 to 90)	304 (179 to 430)	32.1% (18.9%–45.3%)
All form tuberculosis							
Model A	673	335	3210	1600	337 (261 to 414)	1615 (1245 to 1984)	101% (77.7%–126%)
Model B	673	434	3210	2070	239 (202 to 277)	1142 (964 to 1320)	55.2% (46.5%– 63.7%)

See methods for descriptions of models and counterfactual scenarios.

Abbreviations: ACF, active case finding; CNR, case notification rates.

* Relative difference shown for mean CNRs. It is very similar, but not identical, to relative difference in absolute number of diagnoses as underlying population increased over this period.

Under model B assumptions (using non-ACF area as a control), we estimate that, had there been a small increase in tuberculosis diagnoses in ACF areas of equal magnitude to that seen in non-ACF areas, there would have been 199 tuberculosis diagnoses per 100 000 person-years in the 14 quarters between April 2011 and September 2014. Under these assumptions we estimate an absolute increase in mean CNR of 64 tuberculosis diagnoses per 100 000 person-years (95% CI 38 to 90). and a relative increase of 32.1% (95% CI 18.9%–45.3%).

### Trends in All-form Tuberculosis Case Notification Rates in the ACF Area

We also estimated the increase in all forms of tuberculosis, see Table 2 and Figure 3*C* and *D*. Compared to the counterfactual scenario where pre-ACF trends continued (model A, ACF area only), we estimate an absolute increase of 337 all form tuberculosis diagnoses per 100 000 person-years (95% CI 261 to 414) and a relative increase of 101% (95% CI 77.7%–126%). Compared to the counterfactual scenario where trends in ACF area were the same as non-ACF areas, we estimate an absolute increase of 239 all form tuberculosis diagnoses per 100 000 person-years (95% CI 202 to 277) and relative increase of 55.2% (95% CI 46.5% to 63.7%).

## DISCUSSION

The main finding of this analysis is that community-wide ACF was associated with a rapid and marked increase in detection of previously undiagnosed tuberculosis among adults in the central residential neighborhoods of urban Blantyre, Malawi. The direct contribution from microscopy of ACF specimens collected in the field was limited, with only 121 of an estimated 1142 additional people with all forms of tuberculosis (under the more conservative assumptions) directly detected through ACF interventions. Health promotion messages through leafletting, megaphone announcements and conversations with community members may have increased knowledge of tuberculosis and importance of early diagnosis, and contributed to the early impact from our ACF intervention (“indirect effects”) [20]. If so, then an important but often poorly quantified component contributing to the effectiveness of community-wide tuberculosis ACF, is behaviour change with increased care seeking and/or altered health provider behaviour in routine services.

There have been 3 randomized trials assessing the impact of ACF on tuberculosis case notifications in general population [9, 21–23]. An increase in tuberculosis case notifications due to ACF was shown in 1 trial conducted in Ethiopia [22], but no effect of ACF in was seen in 2 trials from Brazil and Ethiopia [21, 23]. These trials had relatively short durations of intervention and follow-up (1–3 years). Another 4 large scale cluster randomised trials have assessed the impact of ACF on tuberculosis prevalence, DETECTB in Zimbabwe [6], ZAMSTAR in South Africa and Zambia [7], ACT3 in Vietnam [8], and TREATS in Zambia [24]—with mixed results. The current study in Blantyre, Malawi, provides unique insight by the long period of follow-up from the first ACF intervention with detailed disaggregated quarterly data, supported by a city-wide surveillance system. Our ACF intervention was relatively “intensive” (eg, more so that Enhanced Case Finding in ZAMSTAR [7]) in terms of frequency of intervention rounds and the door-to-door interactions meaning high amount of contact with community members. However, our ACF diagnostic modality was sputum smear for those with cough, which is not very sensitive (eg, compared to ACT3, which used Xpert for everyone). Presumably more people with TB would have been detected directly through ACF if Xpert had been used, or if chest X-ray was used rather than symptom screening.

We constructed 2 different scenarios to estimate the effect of ACF on CNRs. The first scenario is an interrupted time series within the ACF areas only, showing a marked increase in tuberculosis diagnoses. The limitation of this approach is the relatively short time period prior to ACF introduction and the relatively steep decline in this pre-ACF period. The decline in CNRs between January 2009 and March 2011 pre-ACF might have been due to a true decline in tuberculosis incidence due to scale-up of ART for treatment of HIV in this period [25] but may be related to record keeping or clinician behavior in diagnosing smear negative TB. In second scenario with a control area, we saw an increase in tuberculosis diagnoses at the start of ACF in the non-ACF areas. The most likely cause for at least part of this increase was “spillover” from ACF intervention, both in terms of people in non-ACF areas receiving information about tuberculosis from friends and neighbors in ACF areas, and due to our research team impact in all health facilities raising the “profile” of tuberculosis among healthcare workers by discussions about tuberculosis and by providing sputum culture services. Whether we use the without control (model A) scenario or the with control (model B) scenario, we conclude that there was a substantial impact of ACF on increasing tuberculosis diagnoses.

Sputum microbiological tests for tuberculosis are imperfectly sensitive. Clinical diagnoses are relatively common and often appropriate. However, there is the potential for false positive tuberculosis diagnoses based on clinical diagnosis. False positive tuberculosis diagnoses directly from ACF were not a major concern in this project, as our ACF algorithm only collected sputum from people with tuberculosis symptoms and made diagnoses based on smear microscopy. However, the indirect effects of ACF in prompting healthcare seeking for symptoms that could be tuberculosis could—at least in theory—lead to an increase in false positive tuberculosis diagnoses through clinical diagnoses made by healthcare staff at routine facilities. We collected sputum from everyone starting tuberculosis treatment (whether facility microbiologically confirmed or clinically diagnosed) and despite the large increase in clinically diagnosed tuberculosis in ACF areas, there was no significant difference in ACF and non-ACF areas in the proportion of people whose tuberculosis diagnosis was subsequently microbiologically confirmed indicating that overdiagnosis was unlikely to be the cause of increase in all form TB seen in ACF areas.

The major limitation of this study is the use of tuberculosis CNRs rather than tuberculosis incidence or prevalence [10, 26]. CNRs represent a complex mix of recently acquired and longstanding tuberculosis infection and disease and so can obscure both favourable (reducing tuberculosis prevalence and transmission) and unfavorable (worsening access or reporting) changes in underlying tuberculosis epidemiology and can therefore be difficult to interpret. Although having a control area is a strength of the analysis [19], a limitation is that the control area was not randomly allocated, and the non-ACF areas may not be directly comparable. A further limitation was that the pre-ACF period data were collected retrospectively from clinic paper ledgers, and we were unable to reliably determine age, sex, or HIV status demographics prior to ACF intervention.

Tuberculosis ACF has potential to reduce tuberculosis transmission. In these data from Blantyre, Malawi, we show that interventions combining a period of ACF—alongside with strengthened routine tuberculosis and HIV diagnosis and ART scale-up—can increase tuberculosis case notifications. We are unable to measure whether the increase in CNRs led to a reduction in tuberculosis prevalence. Investigating the extent to which undiagnosed tuberculosis prevalence has fallen through periodic national surveys, or reinstituting surveillance for latent tuberculosis infection and tuberculosis transmission rates will then be critical to assessing whether trends in tuberculosis epidemiology in Malawi are on track to meeting the End tuberculosis Goal of eliminating tuberculosis as a public health problem by 2035. Tuberculosis case notification rates in Blantyre increased concurrently with introducing community based ACF for Tuberculosis, showing potential of ACF to reduce undiagnosed tuberculosis in these communities.

## Supplementary Data

Supplementary materials are available at *Clinical Infectious Diseases* online. Consisting of data provided by the authors to benefit the reader, the posted materials are not copyedited and are the sole responsibility of the authors, so questions or comments should be addressed to the corresponding author.

## Supplementary Material

ciad238_Supplementary_Data

## Notes

***Acknowledgments.*** This project would not have been possible without the contributions of the Laboratory Team, the Field Team, the Data Team, and the Administrators. The authors are also grateful to the Malawi National tuberculosis Programme and the Blantyre District Health Office for their invaluable assistance and partnership.

***Data sharing statement***. All data and code to recreate the analyses are freely available at https://github.com/petermacp/mlwdata (data) and https://github.com/rachaelmburke/tbacf (code and some supplemental data).

***Financial support.*** This work was supported by Wellcome through grants awarded to E. L. C. (grant numbers 091769/Z/10/Z and 200901/Z/16/Z). R. M. B. and P. M. are also supported by Wellcome (grant numbers 203905/Z/16/Z and 206575/Z/17/Z, respectively). P. J. D. was supported by a fellowship from the UK Medical Research Council (grant number MR/P022081/1); this UK funded award is part of the European and Developing Countries Clinical Trials Partnership.
