# Supplementary material for: Impact of Community-Wide Tuberculosis Active Case Finding and Human Immunodeficiency Virus Testing on Tuberculosis Trends in Malawi
Source: Clin Infect Dis. 2023 Apr 26;77(1):94–100. doi: 10.1093/cid/ciad238 (PMC10320183; doi:10.1093/cid/ciad238)
Supplement: ciad238_Supplementary_Data [file ciad238_supplementary_data.pdf]

Supplementary Table 1

People starting TB treatment Q1.2009 to Q1.2011 (pre-ACF)

	ACF (N=1560)	Non-ACF (N=3321)	Total (N=4881)
**How diagnosed**			
a) Clinically dx	936 (60.0%)	2244 (67.6%)	3180 (65.2%)
b) Smr/cult TB lab	0 (0.0%)	0 (0.0%)	0 (0.0%)
c) Xpert clinic	0 (0.0%)	0 (0.0%)	0 (0.0%)
d) Smr clinic	624 (40.0%)	1077 (32.4%)	1701 (34.8%)
e) Direct ACF	0 (0.0%)	0 (0.0%)	0 (0.0%)

People starting TB treatment Q2.2011 to Q3.2014 (during ACF)

	ACF (N=3467)	Non-ACF (N=4822)	Total (N=8289)
**Sex**			
Female	1420 (41.0%)	1918 (39.8%)	3338 (40.3%)
Male	2047 (59.0%)	2904 (60.2%)	4951 (59.7%)
**How diagnosed**			
a) Clinically dx	1754 (50.6%)	2567 (53.2%)	4321 (52.1%)
b) Smr/cult TB lab	437 (12.6%)	587 (12.2%)	1024 (12.4%)
c) Xpert clinic	2 (0.1%)	0 (0.0%)	2 (0.0%)
d) Smr clinic	1157 (33.4%)	1661 (34.4%)	2818 (34.0%)
e) Direct ACF	117 (3.4%)	7 (0.1%)	124 (1.5%)
**Facility**			
a) Central hospital	1302 (37.6%)	2240 (46.5%)	3542 (42.7%)
b) Health centre	928 (26.8%)	743 (15.4%)	1671 (20.2%)
c) Private health facility	223 (6.4%)	286 (5.9%)	509 (6.1%)
d) Not recorded	1014 (29.2%)	1553 (32.2%)	2567 (31.0%)
**HIV status**			
a) HIV-negative	906 (26.1%)	1145 (23.7%)	2051 (24.7%)
b) HIV-positive	2181 (62.9%)	3121 (64.7%)	5302 (64.0%)
c) Not recorded	380 (11.0%)	556 (11.5%)	936 (11.3%)
**ART status**			
N-Miss	1286	1701	2987
a) Not taking ART	780 (35.8%)	996 (31.9%)	1776 (33.5%)
b) Taking ART	1401 (64.2%)	2125 (68.1%)	3526 (66.5%)
**TB Type**			
a) Pulmonary TB	2331 (67.2%)	3264 (67.7%)	5595 (67.5%)
b) Extrapulmonary TB	1136 (32.8%)	1558 (32.3%)	2694 (32.5%)
**Age Group (years)**			
0-14	254 (7.3%)	422 (8.8%)	676 (8.2%)
15-24	472 (13.6%)	643 (13.3%)	1115 (13.5%)
25-34	1294 (37.3%)	1662 (34.5%)	2956 (35.7%)
35-44	871 (25.1%)	1200 (24.9%)	2071 (25.0%)

45-54	307 (8.9%)	546 (11.3%)	853 (10.3%)
55-64	167 (4.8%)	203 (4.2%)	370 (4.5%)
65+	102 (2.9%)	146 (3.0%)	248 (3.0%)

People starting TB treatment Q4.2014 – Q4.2018

	ACF (N=3369)	Non-ACF (N=4852)	Total (N=8221)
**Sex**			
Female	1271 (37.7%)	1818 (37.5%)	3089 (37.6%)
Male	2098 (62.3%)	3034 (62.5%)	5132 (62.4%)
**How diagnosed?**			
a) Clinically dx	1736 (51.5%)	2577 (53.1%)	4313 (52.5%)
b) Smr/cult TB lab	384 (11.4%)	433 (8.9%)	817 (9.9%)
c) Xpert clinic	417 (12.4%)	632 (13.0%)	1049 (12.8%)
d) Smr clinic	832 (24.7%)	1210 (24.9%)	2042 (24.8%)
e) Direct ACF	0 (0.0%)	0 (0.0%)	0 (0.0%)
**Tb type**			
N-Miss	0	3	3
a) Pulmonary TB	2003 (59.5%)	2863 (59.0%)	4866 (59.2%)
b) Extrapulmonary TB	1366 (40.5%)	1986 (41.0%)	3352 (40.8%)
**Facility**			
a) Central hospital	1260 (37.4%)	1478 (30.5%)	2738 (33.3%)
b) Health centre	1012 (30.0%)	1496 (30.8%)	2508 (30.5%)
c) Private health facility	212 (6.3%)	349 (7.2%)	561 (6.8%)
d) Not recorded	885 (26.3%)	1529 (31.5%)	2414 (29.4%)
**HIV Status**			
a) HIV-negative	1046 (31.0%)	1429 (29.5%)	2475 (30.1%)
b) HIV-positive	2235 (66.3%)	3278 (67.6%)	5513 (67.1%)
c) Not recorded	88 (2.6%)	145 (3.0%)	233 (2.8%)
**ART Status**			
N-Miss	1134	1574	2708
a) Not taking ART	216 (9.7%)	389 (11.9%)	605 (11.0%)
b) Taking ART	2019 (90.3%)	2889 (88.1%)	4908 (89.0%)
**Age Group (years)**			
0-14	227 (6.7%)	369 (7.6%)	596 (7.2%)
15-24	412 (12.2%)	621 (12.8%)	1033 (12.6%)
25-34	1066 (31.6%)	1400 (28.9%)	2466 (30.0%)
35-44	1065 (31.6%)	1412 (29.1%)	2477 (30.1%)
45-54	355 (10.5%)	594 (12.2%)	949 (11.5%)
55-64	139 (4.1%)	274 (5.6%)	413 (5.0%)
65+	105 (3.1%)	182 (3.8%)	287 (3.5%)

Supplementary methods for Blantyre Active Case Finding (ACF) intervention

Further details about ACF intervention

The ACF area consisted of three high density urban poor suburbs in North West Blantyre City (Ndirande, Likabula and Chilmoni). The ACF area was divided into 23 neighbourhoods based on road access, natural barriers and community health worker catchment areas, aiming for ~5,000 adults per neighbourhood. Eight briefly trained community workers delivered five rounds of ACF to the total population (one round approximately every eight months) by conducting brief door-to-door visits to each household within the geographically defined zone. Visits were announced the day before by a staff member walking around the community with a handheld megaphone distributing leaflets to anyone interested. A central point was established at a central location, such as a church or school, with a desk and canopy staffed by one team members. The other team members visited each household for a brief door-side enquiry for any adults in the household (present or not) with symptoms of TB, focused but not limited to cough of two weeks or longer. Two Sputum pots and ziplock bags with a pictorial leaflet explaining how to collect sputum effectively were left each person with a reported cough. The households were visited the following morning to collect sputum. Leaflets were left at each household explaining that TB is curable but that untreated TB can remain infectious for years, the important of early TB detection to protect friends and family as well as individual benefits, limitations of microscopy and how to obtain results. Residents could also attend the central point for the duration of the neighbourhood case-finding activities. Positive results were reported directly back to the household with confirmatory specimens and assisted patient registration at the nearest TB treatment facility. Negative results were accessible two to five days after sputum collection at the central hub, but were not reported directly back to patients homes.

Further details about laboratory TB diagnostics

For ACF and tuberculosis registration samples, the tuberculosis research laboratory used double reading of direct auramine-O stained microscopy slides using Primo Star iLED™ microscope (Carl Zeiss Microimaging, Oberkochen, Germany) with confirmation of all positives using Ziehl-Neelsen (ZN) overstaining.

Tuberculosis registration sputum samples (but not ACF microscopy samples) were cultured using BACTEC™ MGIT™ 960 (BD, USA). Mycobacteria species were identified using the MPT 64 antigen test (MGIT TBc Identification test, BD, USA) and microscopic examination for cording.

These laboratory tests were carried out at the TB Research Laboratory at University of Malawi College of Medicine (now Kamuzu University of Health Sciences).

Further details about ART eligibility during study time period

ART was provided by Malawi Department of HIV/AIDS during the time of this study. Eligibility criteria for ART was as follows:[1,2]

2006: ART available for people with CD4 count <250 cells/mm^3 or WHO stage 3 or 4 illness.

2010: ART available for people with CD4 count <350 cells/mm^3 or WHO stage 3 or 4 illness.
First line ART regimen NVP + 3TC + d4T

2011: Option B+ started - lifelong ART started for anyone pregnant or breastfeeding. Otherwise CD4 count <350 cells/mm^3 or WHO stage 3 or 4 illness.
First line ART regimen changed to EFV + 3TC + TDF

2015: Universal treat all – ART eligibility at any CD4 cell count.
First line ART regimen remains EFV + 3TC + TDF

2018: First line ART regimen changed to DTG + 3TC + TDF. All clients on first line ART switched, including those with viral suppression on old regimen.

1. Schramm, B. *et al.* Viral suppression and HIV-1 drug resistance 1 year after pragmatic transitioning to dolutegravir first-line therapy in Malawi: a prospective cohort study. *The Lancet HIV* **9**, e544–e553 (2022).
2. Harries, A. D. *et al.* Act local, think global: how the Malawi experience of scaling up antiretroviral treatment has informed global policy. *BMC Public Health* **16**, 938 (2016).

Blantyre ACF Appendix 2

Pete Dodd

2022-07-22

Mathematical formulation of models

Let $c \in \{1, 2\}$ index the population, with 1 corresponding to the ACF population and 2 to the 'control' population. I think it helps in setting up counterfactuals to keep the population index separate from any indices that control whether ACF is applied.

Let t measure time in quarters from some natural reference point.

Let $\alpha \in \{ACF, notACF\}$ denote whether ACF is applied or not.

Let $P_{c,t}$ be the populations at each time, and let $\pi_{c,t} = \log(P_{c,t})$.

Let $\ell_{c,t,\alpha}$ be the corresponding Poisson rate parameter on a log scale. Let $r_{c,t,\alpha} = \exp(\ell_{c,t,\alpha})$ be the Poisson rate itself.

We will write $\mathbb{I}(t)$ for an indicator function that is 1 during the ACF period, and 0 otherwise, and δ_α for an indicator function that is 1 when $\alpha = ACF$ and 0 otherwise.

Without control

Here, we only have data from the ACF population, and some notation is redundant since this means $c = 1$.

$\ell_{c,t,\alpha} = \pi_{c,t} + k_c + s_c.t + \delta_\alpha \mathbb{I}(t)(a + b.t)$

Here k is the intercept and s the slope, and a and b represent the respective increments to these under ACF. In $\alpha = ACF$ corresponds to the process that gave rise to the data from population 1.

With control

We now want to capture both the intervention effect during the ACF period, and a non-intervention effect during the ACF period. Population 2 allows estimation of the latter. We can separate these out explicitly:

$\ell_{1,t,\alpha} = \pi_{1,t} + k_1 + s.t + \mathbb{I}(t)(\delta_\alpha[a + b.t] + [A + B.t])$

$\ell_{2,t,\alpha} = \pi_{2,t} + k_2 + s.t + \mathbb{I}(t)(A + B.t)$

In fitting to data, $\alpha = ACF$ for $c = 1$ and $\alpha = notACF$ for $c = 2$.

Note: we have restricted the slope (s) in each population prior to the ACF period to be equal due to model fits lacking face validity.

Definition of quantity of interest

Having fitted the models, we want to compute for each the expected cumulative difference in notifications between no-ACF and ACF conditions for the ACF community, $D(\theta)$. This is a function of the model parameters which we will collectively denote θ. That is

$$D(\theta) = \sum_t \mathbb{I}(t)[r_{1,t,ACF} - r_{1,t,noACF}]$$

If $t = t_1$ is the first time in the ACF period, and $t = t_2$ the last,

$$D(\theta) = e^{k_1 + a} \sum_{t=t_1}^{t_2} P_{1,t} \left(e^{(s_1+b).t} - e^{b.t} \right)$$

which has no closed-form answer with the population offset.

For the no-control approach, the corresponding formula is

$$D_{wc}(\theta) = \sum_{t=t_1}^{t_2} P_{1,t} e^{k_1 + s.t} \left(e^{\bar{a}+\bar{b}.t} - e^{A+B.t} \right)$$

where $\bar{a} = a + A$ and $\bar{b} = b + B$.

If the estimate for θ is asymptotically normal with mean $\bar{\theta}$ and variance-covariance matrix Σ, an approximation is that $D(\theta)$ is asymptotically normal with mean $D(\bar{\theta})$ and variance-covariance $J^T \Sigma J$, where J is the gradient (derivative) of D with respect to the parameters θ.

Availability of code

The supplementary data and code files (available at www.github.com/rachaelmburke/tbacf) show the implementation of this work in R.
